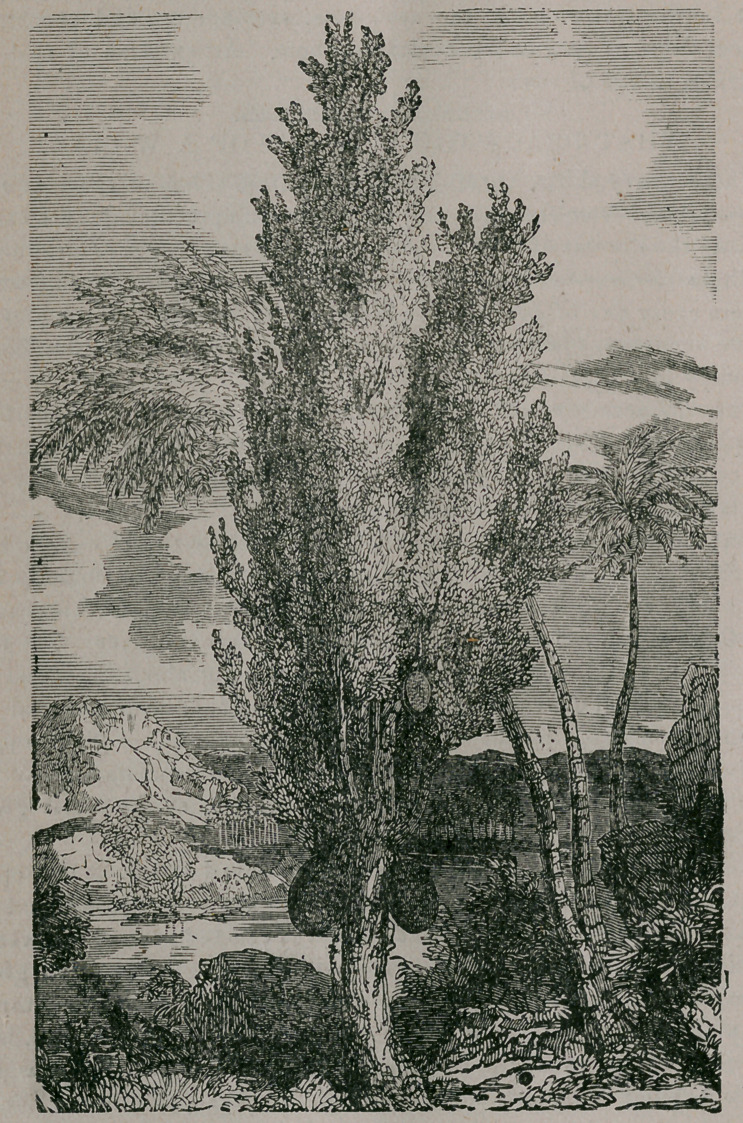# The Jak Tree

**Published:** 1889-07

**Authors:** 


### THE JAK TREE.

The bread-fruit tree, originally found in the south eastern parts of Asia and the islands of the Pacific, though now introduced into the tropical parts of the western continent and the West Indies, is one of the most interesting as well as singular productions of the vegetable kingdom. There are two species of it—the bread-fruit, properly so-called, with the leaves deeply gashed, or divided at the sides, which grow chiefly in the islands, and the jack-fruit, or jak tree, which grows chiefly in the main land of Asia.

The bread fruit is a beautiful as well as a useful tree ; the trunk rises to a height of about forty feet, and in a full-grown tree is from a foot to fifteen inches in diameter ; the bark is ash-colored, full of little chinks, and covered by small knobs ; the inner bark is fibrous, and used in the manufacture of a sort of cloth ; and the wood is smooth, soft, and of a yellow color ; the branches come out in a horizontal manner, the lowest ones about ten or twelve feet from the ground, and they become shorter and shorter as they are nearer the top. The leaves are divided into seven or nine lobes, about eighteen inches or two feet long, and are of a lurid green. The tree bears male and female flowers—the males among the upper leaves and the females at the extremities of the twigs. When full-grown, the fruit is about nine inches long, heart-shaped, of a greenish color, and marked with hexagonal warts formed into facets. The pulp is white, partly farinaceous and partly fibrous, but when quite ripe it becomes yellow and juicy. The whole tree, when in a green state, abounds with a viscid, milky juice, of so tenacious a nature as to be drawn out in threads.

The bread-fruit tree continues productive for about eight months in the year. Such is its abundance that two or three trees will suffice for a man's yearly supply, a store being made into a sour paste called *mahe* in islands, which is eaten during the unproductive season. When the fruit is roasted until the outside is charred, the pulp has a consistency not unlike that of wheaten bread, and the taste is intermediate between that of bread and roasted chestnuts. It is said to be very nourishing, and is prepared in various ways. The jak, or jack, grows to the same or even to a larger size than the bread-fruit of the Society Islands, but it is neither so palatable nor so nutritious. The fruit often weighs more than thirty pounds, and contains two or three hundred seeds, each of them four times as large as an almond. December is the time when the fruit ripens ; it is then eaten, and the seeds or nuts also are eaten, after being roasted. There are many varieties of the jak tree, some of which can hardly be distinguished from the seedling variety of the true bread-fruit. The fruit, and also the part of the tree in which it is produced, vary with the age. When the tree is young, the fruit grows from the twigs ; in middle age it grows from the trunk, and when the tree gets old it grows from the roots.

## Figures and Tables

**Figure f1:**